# Examining purchasing reforms towards universal health coverage by the National Hospital Insurance Fund in Kenya

**DOI:** 10.1186/s12939-019-1116-x

**Published:** 2020-02-03

**Authors:** Rahab Mbau, Evelyn Kabia, Ayako Honda, Kara Hanson, Edwine Barasa

**Affiliations:** 1 Health Economics Research Unit, KEMRI Wellcome Trust Research Programme, P.O. BOX 43640-00100, Nairobi, Kenya; 2 Sophia University, Chiyoda City, Japan; 3 London School of Hygiene and Tropical Medicine, London, UK; 4 Nuffield department of medicine, Oxford University, Oxford, UK

**Keywords:** Universal health coverage, Strategic purchasing, Purchasing reforms, National Hospital Insurance Fund, Equity, Efficiency, Quality

## Abstract

**Background:**

Kenya has prioritized the attainment of universal health coverage (UHC) through the expansion of health insurance coverage by the National Hospital Insurance Fund (NHIF). In 2015, the NHIF introduced reforms in premium contribution rates, benefit packages, and provider payment methods. We examined the influence of these reforms on NHIF’s purchasing practices and their implications for strategic purchasing and health system goals of equity, efficiency and quality.

**Methods:**

We conducted an embedded case study with the NHIF as the case and the reforms as embedded units of analysis. We collected data at the national level and in two purposively selected counties through 41 in-depth interviews with health financing stakeholders, facility managers and frontline providers; 4 focus group discussions with 51 NHIF members; and, document reviews. We analysed the data using a Framework approach.

**Results:**

The new NHIF reforms were characterized by weak purchasing actions. Firstly, the new premium contribution rates were inadequately communicated and unaffordable for certain citizen groups. Secondly, while the new benefit packages were reported to be based on service needs, preferences and values of the population, they were inadequately communicated and unequally distributed across different citizen groups. In addition, the presence of service delivery infrastructure gaps in public healthcare facilities and the pro-urban and pro-private distribution of contracted health facilities compromised delivery of, and access to, these new services. Lastly, the new provider payment methods and rates were considered inadequate, with delayed payments and weak links to financial accountability mechanisms which compromised their ability to incentivize equity, efficiency and quality of healthcare delivery.

**Conclusion:**

While NHIF sought to expand population and service coverage and reduce out-of-pocket payments with the new reforms, weaknesses in the reforms’ design and implementation limited NHIF’s purchasing actions with negative implications for the health system goals of equity, efficiency and quality. For the reforms to accelerate the country’s progress towards UHC, policy makers at the NHIF and, national and county government should make deliberate efforts to align the design and implementation of such reforms with strategic purchasing actions that are aimed at improving health system goals.

## Background

Health financing reforms for universal health coverage (UHC) in low and middle-income countries (LMICs) have mainly focused on two health financing functions: revenue collection and pooling [1]. There is, however, increasing global recognition of the importance of the third healthcare financing function of purchasing as an important policy tool towards achieving UHC [2–4]. UHC emphasizes financial protection and equitable access to good-quality health services according to one’s healthcare needs [5]. Purchasing, which refers to the transfer of pooled resources to healthcare providers for the provision of healthcare services [6, 7], provides a critical link between healthcare financing and healthcare service delivery, and facilitates efficiency, equity and quality in health systems performance [2]. Purchasing can either be passive or strategic. Passive purchasing is the transfer of pooled resources to providers based on historical or predetermined budgets while strategic purchasing involves a deliberate process of determining which services to buy, from who and at what cost with the aim of maximizing health system performance [2, 6, 8].

Kenya, a lower middle-income country, has prioritized the attainment of UHC by 2022 through the expansion of health insurance coverage by the National Hospital Insurance Fund (NHIF) [9]. Current NHIF coverage is 15.8%, which is equivalent to over 80% of the total population with any form of health insurance in Kenya [10]. NHIF is a state corporation whose mandate is to provide health insurance to its members and their dependents [11, 12]. It is one of the healthcare purchasers and the largest health insurer in Kenya with several health insurance schemes (Table 1). Other healthcare purchasers in Kenya include the national government, the county governments, households and private health insurers including community-based health insurers [23, 24].

**Table 1:** Health insurance schemes under the NHIF

Type of scheme	Premium contribution	Population covered
Civil servants scheme^a^ [13, 14]	Mandatory/automatic	Civil servants in the national and county governments, their declared spouse and up to 5 children of up to 21 years of age or 25 years if enrolled in fulltime formal education [13, 14]
County government scheme^a^ [15]	Mandatory/automatic	Employees of County governments that have contract arrangements with the NHIF, their declared spouse and up to 5 children of up to 21 years of age or 25 years if enrolled in fulltime formal education [16]
Parastatal/ private company schemes^a^ [15]	Mandatory/automatic	Employees of parastatals or private firms that have contract arrangements with NHIF, their declared spouse and up to 5 children of up to 21 years of age or 25 years if enrolled in fulltime formal education [16]
National police and Kenya Prisons^a^ [17, 18]	Mandatory/automatic	Kenya Police Force, Administration police, officers in the Criminal Investigations Department, prisons and other security related officers, their declared spouse and up to 5 children up to 21 years of age or 25 years if enrolled in fulltime formal education [16]
Secondary schools’ scheme [EduAfya] [12, 19, 20]	Mandatory/automatic	All students in public secondary schools in Kenya
National scheme [21]	-Mandatory for formal sector workers, -Voluntary for informal sector workers	Any person who is a resident of the republic of Kenya, who is self-employed or in the informal sector, their declared spouse (s) and children up to the age of 18 years
Health Insurance Subsidy for the Poor [HISP] Scheme [22]	Mandatory/automatic	Households with orphans and vulnerable children; poor elderly; and/ or persons, persons with disabilities and destitute families
Linda Mama Free maternity scheme [15]	Mandatory/automatic	All pregnant women who are Kenyan Citizens

^a^ The civil servants scheme, the national police and prisons service scheme, the county government schemes, and the parastatal/ private company schemes form the **enhanced benefits schemes** because they offer comprehensive medical insurance covers [16].

Previous empirical work on purchasing arrangements in Kenya focused on the purchasing practices of the NHIF, county governments, private and community-based health insurers [23–25]. The previous study on NHIF, conducted in 2014, aimed to describe and analyse the purchasing arrangements between NHIF and the government, healthcare providers and citizens [25]. That study, which was conducted before the introduction of the new NHIF reforms described further below, identified significant weaknesses in the purchasing actions between the NHIF as a purchaser and the government, citizens and healthcare providers [25]. For example, along the NHIF-government axis, the absence of an overarching regulatory framework for health service purchasing undermined NHIF’s performance. Along the NHIF-citizen axis, the process for service entitlement design was delinked from citizen preferences, needs and feedback. Along the NHIF-provider axis, weaknesses included inadequate use of quality and efficiency-improvement strategies such as treatment guidelines and generic essential medicines lists [25].

### Recent reforms by the NHIF

In 2015, NHIF introduced several linked reforms aimed at accelerating the country’s progress towards UHC. First, the NHIF revised premium contribution rates upwards (Table 2) with effect from April 1st^,^ 2015 [26]. This was done to cater for the rising cost of healthcare and to enable expansion of its benefits package [27].

**Table 2:** NHIF premiums before and after the 2015 reform in Kenya shillings (KES)/ United States Dollars (USD)

Type of sector	Monthly salary range 1USD = KES 100)	Monthly premium before the reform	Monthly premium after the reform
Formal sector	KES 1000–5999 (USD 10–59)	KES 30–120 (USD 0.3–1.2)	KES 150 (USD 1.5)
	KES 6000–7999 (USD 60–79)	KES 140–160 (USD 1.4–1.6)	KES 300 (USD 3)
	KES 8000–11,999 (USD 80–119)	KES 180–240 (USD 1.8–2.4)	KES 400 (USD 4)
	KES 12,000–14,999 (USD 120–149)	KES 260–300 (USD 2.6–3)	KES 500 (USD 5)
	KES 15,000 and above (USD 150 and above)	KES 320 (USD 3.2)	KES 600–1700 (USD 6–17)
Informal sector (self-employed)	No salary ranges.	KES 160 (USD 1.6)	KES 500 (USD 5)

Second, following the upward revision of the premiums, the NHIF expanded its benefit cover to include outpatient services for the national scheme (previously, the NHIF covered outpatient services for beneficiaries of the enhanced schemes and HISP only) and specialised services for all the NHIF schemes [22]. The specialised services offered include: renal dialysis; kidney transplant; radiology package (magnetic resonance imaging (MRI) and computed tomography (CT)); oncology package (chemotherapy and radiotherapy); chronic illness (diabetes and hypertension); maternity package (normal and caesarean section); rehabilitation package (drug and substance abuse); specialised laboratory tests; surgical package (major, minor and specialised); foreign treatment; and emergency evacuation services [22].

Third, the NHIF introduced new provider payment methods and rates for the new outpatient and specialised benefit packages [22]. It introduced capitation for outpatient services and, case-based and fee-for- service payments for the specialised services. It also revised the per diem rates for inpatient care (already part of the old benefit package) upwards from between KES 600 and 2400 (USD 6–24) [28] to between KES 1500 and 4000 (USD 15–40) per day for the lowest-level and highest-level hospitals, respectively [22]. Table 3 provides a summary of the different payment methods and rates under the NHIF.

**Table 3:** NHIF Provider payment methods and rates [22]

Provider payment method	Healthcare benefit package	Reimbursement rate 1USD = 100 KES	
Capitation	Outpatient services for national scheme members	KES 1200 (USD 12) per beneficiary per year for basic care facilities ^a^(Level 3 and 4)	
		KES 1400 (USD 14) per beneficiary per year for tertiary care facilities ^a^(Level 5 and 6)	
	Outpatient services for civil servants (and other enhanced schemes) of lower job group (A-K or its equivalent)	KES 1500 (USD 15) Public hospitals	
		KES 2850 (USD 28.5) Private hospitals	
Case-based payment	Renal dialysis (pre-dialysis, intra-dialysis and post-dialysis care)	KES 9500 (USD 95) per session twice weekly	
	Kidney transplant package- surgical costs and duration of hospitalization	KES 500000 (USD 5000)	
	Maternity package for National scheme	KES 10000 (USD 100) Normal child birth	
		KES 30000 (USD 300) Caesarean section	
	Maternity package for the Free maternity program	KES 5000 (USD 50) for normal child birth or caesarean section	
	Oncology package- treatment for cancer patients using radiotherapy or chemotherapy case management	Radiotherapy • KES 18000 (USD 180) per week	
		Chemotherapy • Basic- KES 25000 (USD 250) per cycle • Complex- KES 150000 (USD 1500) per cycle	
	Surgical package (covers even cancer surgeries)	Major surgeries • KES 80000 (USD 800) for level 3 and 4 facilities • KES 130000 (USD 1300) for level 5 and 6 facilities Minor surgeries • KES 30000 (USD 300) for level 3 and 4 facilities • KES 40000 (USD 400) for level 5 and 6 facilities Specialised surgeries • KES 500000 (USD 5000)	
	Rehabilitation package (drug and substance abuse)	KES 30000 (USD 300) per year	
Fee-for-service	Radiology package	MRI capped at KES 15000 (USD 150) CT scan capped at KES 8000 (USD 80)	
	Dental cover (Enhanced schemes only) [16]	Capped at KES 50000 (USD 500) per annum	
	Optical cover (Enhanced schemes only) [16]	Capped at KES 40000 (USD 400) per annum	
	Maternity package for Enhanced schemes	Capped at KES 200000 (USD 2000)	
	Inpatient care (medical and surgical conditions that require admission) for Enhanced scheme members of higher job groups (L and above or its equivalent) [14]	Job group or its equivalent	Annual limit
		L	KES 1000000 (USD 10000)
		M	KES 1250000 (USD 12500)
		N	KES 1500000 (USD 15000)
		P	KES 1750000 (USD 17500)
		Q	KES 2000000 (USD 20000)
		R, S, T	KES 2250000 (USD 22500)
	Outpatient care (curative and preventive services) for Enhanced scheme members of higher job groups (L and above or its equivalent) [14]	Job group or its equivalent	Annual limit
		L	KES 100000 (USD 1000)
		M	KES 150000 (USD 1500)
		N	KES 200000 (USD 2000)
		P	KES 225000 (USD 2250)
		Q	KES 250000 (USD 2500)
		R, S, T	KES 350000 (USD 3500)
Per diem	Comprehensive inpatient care (Covers medical and surgical conditions that require admission)	KES 1500–4000 (USD 15–40) for public (government) health facilities and, low- cost private facilities and mission hospitals. No co-payments.	
	Non- comprehensive inpatient care (Covers medical and surgical conditions that require admission)	KES 2000–4000 (USD 20–40) for high-end private hospitals. Members pay top up deficit by self-pay or co-insurance	
	Foreign treatment	KES 1700 (USD 17) per day of hospitalization	

^a^Level 3- Offer basic outpatient, basic maternity (obstetric) services and routine laboratory tests

Level 4- 1st referral hospitals. Offer a broader range of inpatient and outpatient care, emergency obstetric care, specialised laboratory and radiology services.

Level 5- 2nd referral hospitals. Offer more comprehensive specialised outpatient and inpatient curative services including intensive care.

Level 6- Highest level of care in the Kenyan health system. Offer highly specialised and complete set of care.

These three purchasing reforms are UHC-inspired reforms as they seek to expand the benefit package and population covered while reducing the costs of out-of-pocket (OOP) payments associated with the use of these services [29] with important implications for the health systems goals of equity, efficiency and quality [30]. These reforms, separately and together, have the potential to influence the purchasing practices of the NHIF. The aim of this study is to examine the influence of the purchasing reforms on NHIF’s purchasing practices and the implications of this for strategic purchasing and health system goals of equity, efficiency and quality.

## Methods

### Study design

We employed an embedded case study design [31] with the NHIF as the case and the different reforms as the embedded units of analysis. A case study is an empirical enquiry that investigates a real-life phenomenon through a detailed contextual analysis of the phenomenon [31]. A case study approach is appropriate for this study because it provides a structured yet flexible approach to data collection and analysis using multiple sources of evidence [31, 32].

### Study framework

**Fig. 1:**
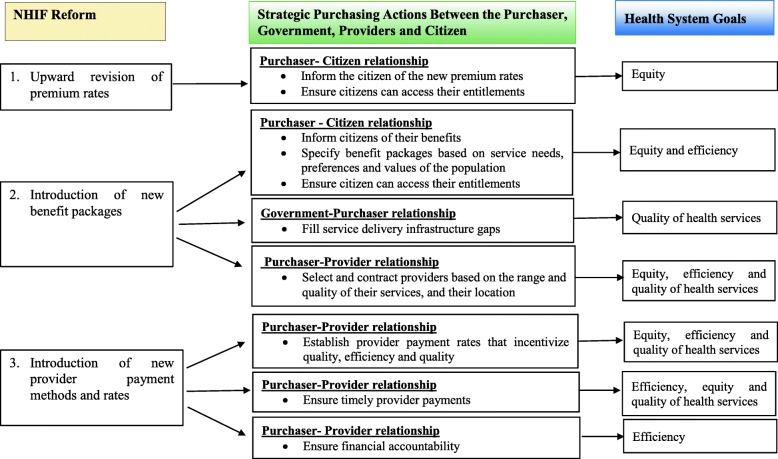
Conceptual framework

We applied the Resilient and Responsive Health Systems (RESYST) conceptual framework for purchasing (Fig. 1). This framework outlines the key strategic purchasing actions that characterise the three purchaser relationships: Government and purchasers, purchasers and healthcare providers, and citizens and purchasers [2]. For this study, we adapted the RESYST conceptual framework by including the NHIF’s purchasing reforms and potential implications of the reforms on the health systems goals mediated through the key purchasing actions (Fig. 1). In this study, the purchaser is the NHIF while the 1) government, 2) providers and 3) citizens are represented by the 1) Ministry of Health (MOH) (national government) and County Department of Health (county government), 2) public and private health facilities and, 3) NHIF beneficiaries respectively. This study specifically focused on the effect of the NHIF purchasing reforms on the strategic purchasing actions involved in each of the key relationships and the implications of this on health system goals.

### Study sites

We purposively selected respondents with knowledge of and experiences with NHIF since this was the phenomenon of interest in our study [33]. We selected participants at the national and county level. Participants at the national level included health financing stakeholders (policy makers, implementers) and development organizations providing technical and/or financial support to health financing initiatives in Kenya. These participants included officials from MOH, NHIF and development partners. At the county level, we selected two counties (Table 4) with contrasting levels of socioeconomic factors, health indicators, health sector resource allocation (a proxy indicator of the importance attached to the health sector by the respective county), logistical reasons and safety assurance for the researchers. We have anonymized the counties to maintain confidentiality of the study participants.

**Table 4:** Characteristics of study counties

Characteristic		County I	County II	Kenya
Projected population 2015		1,831,800	1,107,755	44,157,000
Unemployment rate		19%	12.5%	7.4%
Poverty rate		21.8%	60%	36.1%
Distribution of health facilities by ownership	Private (for-profit and not-for-profit)	80.5%	45%	50.3%
	Public	19.5%	55%	49.7%
Doctor: population ratio		1:17000	1:44634	1:10000
Immunization coverage		90%	53.6%	80%
Percentage of County budget allocated to health (Financial Year) FY 2015/16		32%	22%	Average percentage of county budget allocated to health FY 2015/16 = 23.4% *Target = 35%
Percentage of County budget allocated to health FY 2016/17		33%	27%	Average percentage of county budget allocated to health FY 2016/17 = 25.2%

In each county, we selected three NHIF accredited health facilities that could offer services under the new benefit packages (that is, outpatient, inpatient and specialised health services): one public first referral (Level 4), one public second referral (Level 5) and one private (level 4) facility. Permission to conduct the study in these facilities was provided by the respective institutional heads. Table 5 outlines the characteristics of the selected hospitals which have been anonymized to maintain confidentiality of the study participants.

**Table 5:** Characteristics of study hospitals

Characteristic	Hospital A	Hospital B	Hospital C	Hospital D	Hospital E	Hospital F
Ownership	Public	Public	Public	Public	Private	Private
Level	Level 4	Level 4	Level 5	Level 5	Level 4	Level 4
County	County I	County II	County I	County II	County I	County II
Total annual outpatient attendance workload	294,352	121,661	330,022	283,677	19,153	18,539
Total annual inpatient admissions	20,534	11,593	22,013	22,622	3366	1984
Number of beds	289	195	457	265	20	36

### Data collection

We collected data between September and December 2017 through in-depth interviews (IDIs), focus group discussions (FGDs) and document reviews. All the participants included in this study gave written informed consent after being presented with information on the organisation conducting the study, why the study was being done and who the researchers were. Two researchers (EK and RM) conducted 41 IDIs in English with participants from the national and county levels (Table 6) using semi-structured interview guides developed from the study’s conceptual framework (Fig. 1). First, we developed interview questions specific to each reform using the relevant strategic purchasing actions as outlined in the framework. Then we developed probes relating to equity, efficiency and quality based on broader reading of literature (See Additional file 1: Semi-structured interview guide). The trustworthiness or construct validity of the semi-structured interview guide was tested by a team of health economics researchers in our research organization in Kenya who checked for ambiguities and leading questions. All IDIs were conducted at the participant’s workplace and were audio-recorded using encrypted audio-recorders with consent from the participants. Each IDI lasted between 35 and 70 min.

**Table 6:** Summary of study respondents

National level respondents					
	Male		Female		Total
Ministry of Health	1				1
NHIF	1		1		2
Development partners	3		1		4
Total national level participants	5		2		7
	**County I**		**County II**		**Number**
County level participants	**Male**	**Female**	**Male**	**Female**	**Total**
County health department officials (County director of health, County nursing officers, county clinical officers)	1	1	1	2	5
NHIF branch officials	2	1	1	1	5
Public hospital managers (Medical superintendent, Nursing services managers, Pharmacist in-charge, Clinical officer in-charge)	1	2	2	2	7
Private hospital managers (Medical managers, Nursing services managers)	1	1	–	1	3
Public hospital frontline health workers (Clinical officers, Nurses)	1	1	1	1	4
Private hospital frontline health workers (Clinical officers, Nurses, pharmacists)	1	2	1	2	6
Public hospital NHIF billing clerks	–	1	1	–	2
Private hospital accounts staff	1		–	1	2
Total county level participants	8	9	7	10	34

Two researchers (EK and RM) conducted 4 FGDs with 51 NHIF members- 2 FGDs in each county included in the study. Each FGD comprised of a total of 10–16 NHIF members, both men and women of various ages and employment status (formal and informal) as identified by regional NHIF officials and local community health volunteers. Table 7 provides a summary of these characteristics. FGDs were conducted at a location central to the participants. All FGDs were recorded in Swahili using encrypted audio-recorders with consent from the participants and lasted between 60 and 90 min. Swahili was chosen as the medium of communication for the FGDs since community participants had varied levels of education and hence varied levels of competency in English but were all competent in Swahili. The interviewers, EK and RM, were conversant with Swahili which is Kenya’s national language. Three researchers (EK, RM and EB) held face-to-face peer debriefing sessions after conducting IDIs and FGDS to critique the data collection process, identify areas that needed further probing and to discuss the emerging themes [34]. We stopped data collection once saturation- point of no new information [35]- was reached in both the IDIs and the FGDs.

**Table 7:** Characteristics of FGD participants

Attribute	County I		County II	
	FGD I	FGD II	FGD I	FGD II
Gender	Male (*n*) = 6	Male (*n*) = 7	Male (*n*) =4	Male (*n*) = 5
	Female (*n*) = 6	Female (*n*) = 9	Female (*n*) = 6	Female (*n*) = 8
Age range	28–57	31–65	25–53	33–67
Employment status	Formal sector (*n*) = 9	Formal sector (*n*) =12	Formal sector (*n*) = 8	Formal sector (*n*) = 10
	Informal sector (*n*) = 3	Informal sector (*n*) = 4	Informal sector (*n*) = 2	Informal sector (*n*) = 3
Total FGD participants (*n* = 51)	County I (*n*) = 28		County II (*n*) = 23	

Lastly, we reviewed policy documents (Table 8), regulations, press releases, NHIF websites, grey and peer reviewed literature for information regarding the new reforms, their effect on NHIF’s purchasing actions and health system goals of equity, efficiency and quality of health services.

**Table 8:** Documents included in the review

National statutory documents	The Constitution of Kenya
	Vision 2030
	Kenya Household Health Expenditure and Utilisation Survey.
	Kenya Service Availability and Readiness Assessment Mapping (SARAM) Report.
	Mini-service availability and readiness assessment (MINI-SARA) 2016 survey report
	National Hospital Insurance Fund Act
NHIF documents	Annual Management report 2015/16
	Annual Management report 2016/17
	Explanation of the benefit package for the National Scheme.
	NHIF report on availability and quality of NHIF services at the healthcare provider and NHIF offices
	Comprehensive medical insurance scheme for civil servants & disciplined services hand book
	Handbook For Provision Of Comprehensive Medical Cover, Group Life Insurance and Last Expense Cover to Civil Servants
	Enhanced Benefits Medical Scheme Book
	National Police service & Kenya Prisons Service Comprehensive Medical Cover & Last Expense Handbook

### Data management and analysis

All audio records from the IDIs were transcribed verbatim in English. FGDs were transcribed verbatim in Swahili and translated into English. All transcripts were reviewed for transcription accuracy by comparing them against their respective audio files. The transcripts were then imported into QSR NVIVO 10 [36] to manage coding and analysis. We used a Framework approach to analyse data. The Framework approach is an analytic process that involves a systematic process of sifting, sorting, coding and charting data into key issues and themes [37]. One researcher (RM) first familiarized herself with the data through line-by-line reading and re-reading of the transcripts. She then developed codes deductively from the study’s conceptual framework and applied the codes to segments in the transcripts that were interpreted as important. All the study team members (AH, EB, EK, KH and RM) then reviewed and discussed the initial coding framework and coded data. Discrepancies in coding were discussed and reconciled appropriately before the final coding framework was approved by the study team. Two researchers [EB and RM] then applied the final coding framework to the rest of the data grouping similar codes into themes and later charted the data to allow development of meaning through comparisons and interpretations.

## Results

The results are grouped in relation to each NHIF reform, and within each reform according to the relevant purchasing relationships and actions (see Fig. 1).

### NHIF reform 1: upward revision of premium contribution rates

#### Purchaser-citizen relationship

##### Inadequate communication on the upward revision of premium contribution rates

The NHIF employed various mass communication media to inform members of the public of the new monthly premium contribution rates, their timing, how to remit them and penalties for late payments. The channels included: television, radio, newspapers, pamphlets, the NHIF website, social media, billboards, mobile phones (short message service), and sensitization campaigns at both national and county levels. Study participants, however, felt that communication through these channels did not reach some key population groups such as the elderly, the uneducated, the unemployed, people living with disabilities (visual or hearing disabilities), the poor and the people in the rural and marginalised areas. This was blamed on limited access to these media platforms as well as limited number of NHIF service points.

*“Those people in remote areas have not been reached especially where NHIF offices cover a large area. They cannot give that information immediately because it takes time for them to go and access those people and give that information”* Male participant, NHIF, County I

These population groups were less likely to be aware of the new premium rates. They continued making premium payments using old rates which limited their access to the new benefit entitlements since they were no longer considered active contributors.

*“At the village, some people are not aware at all of the increased premium rates and how they operate. It is only when they go to the hospital that they really get shocked that they cannot get services and they are being told to go to the NHIF office.”* Male Participant, FGD I, County II

##### Unaffordability of the increased premium rates limited access of certain citizen groups to the entitlements

**Fig. 2:**
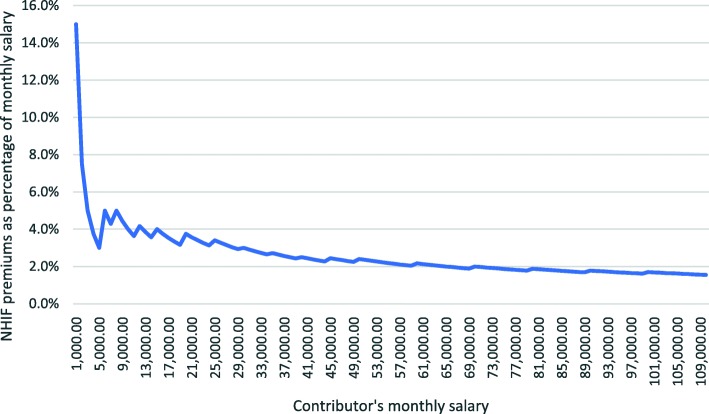
Regressivity of premium contribution rates after the reforms

Study participants considered the new premium rates for the informal sector unfair and unaffordable as they were fixed over quite large income ranges. Premium contributions for the formal sector were however graduated depending on the salary range. Our review of the new premiums rates showed that they were regressive as low-income earning population groups in both the formal and informal sectors contributed more of their income towards the premiums than higher-income earning groups (Fig. 2).

Unaffordability of the new premiums was considered a barrier to enrolment and hence a barrier to access to needed care particularly for the unemployed; those living in rural and marginalised areas; the youth of 18 years and above but not enrolled in school; the elderly; people living with disabilities and those in the informal sector with meagre and unstable earnings.

*“In the village, there are youths who have finished school without formal employment but their age surpasses the required age for the cover under their parents. As a parent getting that 500 shillings to pay for him or her is a problem. They are not covered.”* Female participant, FGD II, County II

*“There are quite a number of people who are not taken care of like the disabled group who cannot afford to pay for the national scheme. And where do they fall? They also become sick. Unless someone pays the money for them”* Male manager, private hospital F

FGD participants working in the informal sector cited the higher premium rates as a cause of attrition as they could no longer afford them. Document reviews showed that while total NHIF membership had grown from 4 million in 2012 to 6.8 million in 2017, only 48% of these members were active contributors [15]. Active contributors were fewer in the informal sector than formal sector. Only 27% of informal sector members had active membership compared to 65% of the formal sector members as of June 2017 [15].

*“Now we are required to pay 500 shillings. This is too much money for poor people like me. I have nowhere to get that kind of money. I felt that I had no option but to abandon my card”* Female participant, FGD II, County I

*“NHIF is for those who can afford”* Male participant, Development partner, National-level

### NHIF reforms 2: introduction of new benefit packages

#### Purchaser-citizen relationship

##### Inadequate communication on the new benefit packages

The NHIF used the same channels used for communicating the new premium rates to provide information on the new benefit packages including how and where to access them. To access outpatient services, NHIF members and their dependents were expected to pre-select a health facility of their choice [38, 39] by completing a choice of outpatient medical facility form [40]. On the other hand, pre-selection was not required for NHIF members and their dependents to access specialised services. Just as with communication of the new premium rates, study participants felt that the elderly, uneducated, unemployed, people living with disabilities, poor and people in the rural and marginalised areas were less aware of the new benefits or where and how to access them. The lack of awareness limited their access to and utilisation of these new services. Document review showed that there was low enrolment for outpatient services with only 21% of the registered members having selected their preferred healthcare providers [22]. This was partly attributed to poor communication of the access requirements to the NHIF beneficiaries [22].

*“They are not fully aware because some people will say ‘I have registered under NHIF’ but they do not know there is something called capitation and there are restrictions with capitation. You only go where you are capitated. They must be told categorically that with NHIF, they and their dependents must be capitated somewhere to access the outpatient services. They also do not know that for inpatient care, they can go anywhere. Even those who are capitated in this hospital think that they must come for admission in this hospital not knowing that they can also be admitted anywhere else as long as they have the cover and that they meet the category of the hospital.”* Male manager, Hospital F, County II

Some FGD members and development partners felt that knowledge and awareness of the new reforms by certain population groups was limited by: 1) the use of complex language (such as medical terms) to describe the services offered, 2) vagueness of the benefit package where services were presented in broad categories making it difficult for users to know what specific services they are entitled to, 3) limited geographical coverage of communication and sensitization campaigns and, 4) limited knowledge and awareness among some healthcare providers who were gatekeepers for access to these services.

*“There is a problem in NHIF. They have not done communication to all health facilities that we have registered with. Sometimes you are chased away and they do not normally honour us”* Male participant, FGD I, County II

##### The benefit packages were based on service needs, preferences and values of the population

The choice of services included in the new benefit packages was informed by population health needs and preferences based on disease burden from the claims received at the NHIF office, national disease burden as indicated in national reports and strategies, and complaints and preferences provided by patient support groups and NHIF beneficiaries themselves.

*“We knew the needs of our members. Some of the biggest health problems were oncology and surgeries. There was also this study on non-communicable diseases that was done by the Ministry that showed that cancers, diabetes and hypertension were on the rise. We also considered information from our data, and from complaints and requests received from our members through their own groups like the Kidney care group which pushed us to include dialysis sessions.”* Female participant, NHIF, National level

Frontline providers and FGD participants also felt that the services offered under the new benefit packages addressed prevailing disease burden.

*“Yes, people’s needs are covered. Majority of our patients come here with typhoid and malaria which are covered. If somebody has hernia or needs a caesarean section, surgery can be done. Normal deliveries are also covered. If somebody needs laboratory tests, they can be done. Most of the important things that the patients need are covered.”* Female frontline provider, private hospital F

*“The advantage lies in the fact that we can now get treatment for life threatening ailments such as cancer, cardiovascular disease, diabetes and others”* Male participant, FGD I, County I

##### Unequal distribution of entitlements in the new benefit packages across different population groups limited citizen’s access to the entitlements

While the new benefit packages were responsive to the needs of the citizens, study participants reported that civil servants and other members of the enhanced schemes received a wider range of services compared to members of the National scheme and HISP. A comparison of the benefit packages across the different NHIF schemes from document reviews showed that beneficiaries of the National scheme and HISP were not entitled to preventive health services and a few other curative services that beneficiaries of enhanced schemes receive (Appendix). These differences led beneficiaries of the National scheme to make additional OOP payments to access needed services.

*“Late last year, my mother was hospitalized at the district hospital. We bought medicines daily. When we went to collect the prescribed medicines in the pharmacy, there were some classifications. There were those who were given medicines but told to pay more and those with an enhanced scheme who got medicines for free. This disparity brought a lot of problems because you see your next neighbour, who is a patient like you, being served while you cannot get the same medicines without buying them.”* Female participant, FGD I, County II

#### Government- purchaser relationship

##### Presence of service delivery infrastructure gaps for the new benefit packages in public healthcare facilities

While the new reforms included new benefit entitlements, they were not accompanied by reforms on infrastructure improvement. Study participants reported that contracted public hospitals, particularly in rural and marginalised areas, lacked appropriate service delivery infrastructure for the outpatient and special benefit packages in terms of medical equipment, medicines and human resources (medical officers, specialists and nurses). A review of the service availability and readiness assessment reports supported these claims as they highlighted the limited availability of: 1) essential medicines [41], 2) laboratory supplies [42] and, 3) specialised services such as dialysis, chemotherapy, radiotherapy and organ transplants [42] in public hospitals.

*“There is a shortage of doctors and specialists like oncologists. Public hospitals are also overstretched and they do not have tests such as CT Scan or ultrasound which forces you to seek care in a private hospital. So, in as much as you have an NHIF card, it is not very useful in a public hospital because you will not get the services.”* Female participant, FGD II, County I

#### Purchaser- provider relationship

##### Selection and contracting of providers was characterized by a pro-urban and pro-private distribution

To increase access to the new benefit packages, the NHIF embarked on a drive to expand the network of healthcare facilities to contract with. Between 2013 and 2017, the number of contracted healthcare facilities (both public and private) increased from 1237 to 5258. Study respondents reported that most of the contracted health facilities within their counties were found in the urban and peri-urban areas, particularly private hospitals, which created greater geographical inequalities in access for the people living in rural and marginalised areas.

*“NHIF historically was only covering hospitals and we know that in terms of facility density, most of the facilities in rural areas are primary level so already there is a disproportion there. Even with the hospitals, it was a specific set of them. Not all hospitals were NHIF accredited. Now they have started going to the primary level facilities but not all of them have been brought on board. Therefore, access to care in rural areas is much lower than in urban centres and we know that the larger proportion of our population lives in rural areas.”* Female participant, Development partner, National-level

Document review showed that private health facilities (both private-for-profit and private-not-for-profit) accounted for 75.5 and 56.9% of all health facilities contracted by NHIF in County I and II, respectively. While this finding may be expected for county I which has a higher proportion of private health facilities, it was not expected for county II which had a higher proportion of public than private health facilities. NHIF officials felt that this resulted from a lack of initiative by government health facilities to seek contracts with NHIF as well as staff shortages within NHIF to do active follow up on the same.

*“Private hospitals are more. It is only when we started the government initiative of Linda mama [free maternity programme] that we brought in so many government health facilities. However, it is not easy to bring them on board because they are not motivated even to come here. It is us to communicate with them, call them for letters or deliver those letters to them which is not easy. But for private facilities, it is a self-initiative.”* Female participant, NHIF branch official, County II

FGD participants felt that the pro-private distribution of contracted health facilities within their counties was discriminatory because it was mainly civil servants who could access services in these facilities. Access to the health facilities varied by type of scheme. Members of the national scheme accessed services from contracted public facilities or low-cost private hospitals. Civil servants and members of enhanced schemes could access both contracted public and private health facilities including high cost private hospitals [14, 16, 18]. In addition, FGD participants felt that services offered in private-for-profit hospitals were more expensive than in the public hospitals which put them at risk of making additional OOP payments or depleting the limits capped on specialized services by the NHIF.

*“I took my son to a private hospital where he was diagnosed with ulcers caused by H. pylori. His medicines cost almost 14,000 shillings. I panicked because I knew we would exhaust our yearly allocations. I also developed the same problem but I went to the Level 5 public hospital. The treatment did not cost me more than 800 shillings. I even argued with the doctor about this. How comes my son’s H. pylori kits cost 14,000 in the private hospital but it cost me less than a thousand shillings in a public hospital? This is very bad”* Male participant, FGD I, County I

### NHIF reforms 3: introduction of new provider payment methods and rates

#### Purchaser- provider relationship

##### The new provider payments did not incentivize equity, efficiency and quality healthcare service provision due to perceived inadequacy in payment rates

Both public and private providers indicated that the capitation rates offered for outpatient services were inadequate as they did not take into consideration the actual costs of services or the number of times an NHIF beneficiary would visit a health facility. NHIF officials reported that the capitation rate was informed by consultations with bodies representing both public and private providers and, findings from actuarial analysis and costing studies. However, officials from development partners, county department of health and county health facilities reported not being involved or even aware of any actuarial costing studies.

*“I remember being in those discussions a while back where we were asking NHIF officials what the basis for the capitation was. They kept saying that they had done some costing studies but they never shared any of that information. We wanted to know the basis of going from 2,850 shillings for the civil servants to 1,200 shillings for the national scheme. I mean at least for the reimbursements for the special packages they could go and observe what the market rates are, right?”* Female participant, Development partner, National-level

The perceived inadequacy of capitation incentivized some public providers to ask patients, particularly those with chronic illness, to buy medication elsewhere. For private providers, it incentivised them to either undertreat, charge co-payments, refer to other accredited health facilities or, admit NHIF beneficiaries who would have otherwise just required outpatient care. Media reports indicated that major private hospitals had altogether rejected capitation which further limited access to care for members of the national scheme [43, 44]. Healthcare providers did not understand that capitation refers to a fixed amount of money paid per patient per unit of time based on population level data on costs and average utilization of services.

*“For fee-for-service, we can do more laboratory tests compared to the national scheme. For national scheme, I am told not to send them to the laboratory for many investigations because their capitation money will be exhausted just from consultation without even medication. So, I am forced to treat clinically yet some patients require a conclusive test that will help us manage them well. Yes, we are forced to cut on cost.”* Male frontline provider, private hospital F

##### Provider payments were not timely

According to public and private hospital managers, the NHIF did not make timely payments to providers for the provision of services outlined in its benefit packages including the new outpatient and specialised services. In policy, capitation for outpatient services was to be paid before service delivery (at the beginning of each fiscal quarter) while payments for inpatient and specialised services were to be reimbursed within 14 working days following submission of claims data [45]. In practice however, NHIF disbursed capitation in the middle of the fiscal quarter and took between 2 and 3 months to honour and reimburse claims for inpatient and specialised services. This was attributed to the manual claim process that was demanding, the limited number of NHIF claims and benefits officers, and reports of limited financial resources within the NHIF.

*“So even claims that we took two months ago have not been reimbursed because they are down financially. The NHIF informed us that they do not have money”* Male manager, public hospital C, County II

NHIF beneficiaries seeking care in private facilities felt that the delayed reimbursements by NHIF had incentivized providers to: 1) introduce co-payments, 2) deny or ration services offered to them, 3) treat them with less respect, or 4) expose them to longer waiting times than patients with other forms of insurance or cash paying patients.

*“Private hospitals do not treat us with respect. There is even a notice, “do not give these medicines to NHIF patients” in their pharmacy. If you peep inside, you will see the label. It is as if you are a second-class citizen from those with other medical covers who are treated superiorly.”* Female participant, FGD II, County II

*“It is like torture there because those who have cash are treated quickly. So, at times when I go to the hospital, I feel that it is better for me to use cash than to use my NHIF card so that I can get treatment faster.”* Female participant, FGD II, County I

##### Weak financial accountability led to fraud by healthcare providers, NHIF officials and NHIF beneficiaries

Respondents reported various cases of fraudulent behaviour for financial gains by healthcare workers, NHIF officials and NHIF beneficiaries- a finding that was supported by various media reports [46–48].

*“There has been massive fraud. Some of the providers are working with people at the NHIF to defraud NHIF and one of the areas they have been taking advantage of is the issue of major surgery and minor surgery. Most hospitals have been converting the minor surgeries to major surgeries so that they can get higher reimbursement. The fraud has been there but it has become a big issue since the introduction of those specialised packages”* Male participant, Development partner, national-level

Several system weaknesses had led to this. First, the self-assessment process- whereby health facilities could assess their own structural capacity prior to contracting [49]- created a loophole for fraudulent behaviour. County officials reported that some providers exaggerated their structural capacity (e.g. beds, medical equipment and theatre rooms) during this process to obtain a higher level of reimbursements from per diem and case-based payments for inpatient and specialised services respectively.

*“When we looked at the facility, they did not have a theatre yet NHIF said they were making claims for operations done. So how did they do the operations without the theatre? It was questionable. There was a room that they said was a theatre but we know that a theatre is not just a room. The facility did not have all the things required to make a theatre”* Male county level manager, County I

Secondly, the limited number of NHIF officials (claims and benefits officers, and quality assurance officers) undermined frequent surveillance of health facilities. Their limited number coupled with the manual claim confirmation and reimbursement process and increased workload, created a barrier to a fast and efficient process of confirming the authenticity of claims.

“*Surveillance should be done at least twice a week but we face challenges. There are many contracted health facilities and fewer quality officers so it is not easy for us to reach all of them. So now we do what we call smart surveillance- where we visit health facilities with high claim notifications”* Female NHIF branch official, County II

Thirdly, weak identification processes for NHIF beneficiaries seeking care incentivized some beneficiaries to engage in fraudulent behaviour such as: 1) use of false identity cards to obtain care, 2) hiring out of the NHIF cards to those without but who needed access to care, 3) use of NHIF cards to illegally obtain medication for people who were not members of the scheme or, 4) impersonation.

*“The challenge we have in government (public) facilities is actually that of fraud through impersonation where one gives their card to their sibling to pass as them in order to use the card to access services”* Male NHIF branch official, County I

NHIF officials and media reports indicated that the NHIF was however implementing various strategies to curb fraudulent activities such as: online notifications for inpatient admissions, centralised authorisation (letters of undertaking) for surgical procedures and specialised imaging studies [50] and, introduction of biometrics for identification of beneficiaries.

*“We are going the biometric way. We assessed and provided some health facilities with the gadgets and software. Once we have all the biometric features for the members, we will reduce cases of impersonation and cases of patients not being in the wards when they were supposedly admitted.”* Male NHIF branch official, County II

## Discussion

In this study, we set out to examine how the recent reforms of the NHIF affected its purchasing practices. Our study findings show that even with the new reforms, the NHIF remains a passive purchaser with negative implications for equity, efficiency and quality arising from weaknesses in either the design or implementation of the reforms.

### Equity implications

First, the higher premium rates were unaffordable and regressive for the poor, elderly, people living with disabilities, unemployed and informal sector workers with meagre and unstable earnings. This policy design created a financial barrier to enrolment and led to attrition. Affordability of premium contributions has been shown to affect enrolment and retention in other LMIC settings such as Ghana [51–53], Uganda [54], Burkina Faso [55] and Nigeria [56]. In Ghana, informal sector contributions were found to be particularly regressive [57]. Second, differences in the benefit package between the national scheme and enhanced schemes, a policy design issue, led to inequities in access to services and OOP payments for the services not covered. Differences in benefit package design lead to differences in financial protection where generous benefit packages are associated with lower OOP payments [58, 59]. Third, limited awareness of the new benefits and premiums by the poor, elderly, unemployed, informal sector workers, people in the rural areas and those with disabilities due to varying access to mass media and NHIF service points, a policy implementation issue, led to variations in enrolment, access to care and unnecessary OOP payments. Studies in Nigeria, Burkina Faso, Kenya and Ghana have shown that awareness of insurance schemes affects participation and enrolment [56, 60–62] as well as service utilisation among enrolees as seen in India [63]. A guiding principle for purchasers is that communication of UHC-inspired reforms should inform the population of their entitlements and obligations [2, 64] through public provision of detailed but simplified information on included and excluded services and the associated levels of OOPs [30, 65]. This empowers beneficiaries, particularly marginalised groups, to claim their benefits and entitlements and improves utilisation among these population groups [30]. It also provides one of the best strategies to reduce possible variations in access to care [65] and informal or OOP payments by beneficiaries to providers [65, 66]. A fourth factor undermining equity was the pro-urban and pro-private distribution of contracted health facilities. This policy implementation issue undermined access for the poor and those living in rural and marginalised areas who are known to have higher burden of disease and limited financial protection [67] which predisposes them to catastrophic health expenditures [68]. In Kenya, 65.3% of rural populations depend on public health facilities for outpatient services [69]. In fact, private hospitals (both for-profit and not-for-profit) have been shown to be pro-rich [70]. Decisions to increase coverage should therefore give priority to these groups [4, 30] and to the public sector by ensuring that it is well-funded and well- structured with adequate service delivery infrastructure [4, 71]. Lastly, perceived inadequacy of, and delayed NHIF reimbursements, a policy implementation issue, led to preferential treatment of privately insured and/ or uninsured cash-paying patients over NHIF beneficiaries particularly in private hospitals. The provider incentives embodied in payments systems influence provider behaviour in treatment decisions which in turn affect equity in access to needed services, quality and efficiency of service provision [8, 72].

### Efficiency implications

We identified two main implications for efficiency. First, unnecessary admissions and referrals due to perceived inadequacy of capitation rates, a policy design and implementation issue, compromised efficiency since resources were spent on unnecessary care. Second, weak accountability mechanisms, a policy implementation issue, compromised efficiency through loss of resources to fraudulent activities. Unnecessary hospital admissions and fraud are among the top ten causes of inefficiency in health systems globally [3].

### Quality implications

Quality of care particularly in public hospitals was compromised by lack of accompanying reforms on quality and infrastructure improvement- a weakness in the design and implementation of the new policies. High quality care requires skilled health workers, well-equipped hospitals and reliable medicines [73] that are equitably distributed to promote equitable access to services in the benefit package [5]. Reforms of benefit packages should also inform infrastructure developments [30, 65], failure to which makes the benefit package merely a wish list, with limited access to actual services and limited financial risk protection [65]. Quality was also compromised by rationing of services to patients due to perceived low provider payment rates and delayed reimbursements which are both weaknesses in the design and implementation of the new policies.

## Recommendations

Drawing from these findings, we recommend that policy makers at the national and county governments as well as the NHIF, address weaknesses in the design and implementation of the reforms for them to be successful.

The National and county governments should improve the infrastructural capacity of public healthcare facilities to support the UHC- inspired NHIF reforms. This includes priority attention to human resource for health, medicines, and medical equipment. They should also find innovative ways of financing premiums for the poor, elderly, people with disabilities, unemployed and those in the informal sector in a bid to leave no one behind. This can be done through specific allocation of general government revenue.

For its part, the NHIF should first, re-orient its facility selection to create a balance between public and private facilities, and between urban and rural facilities to improve geographical access. Second, engage healthcare providers in determining provider payment rates and publicly avail information on how the rates are developed. This will improve provider acceptance. Third, actively educate health workers on the services offered in the benefit package as they are the gatekeepers who provide access to health services. Fourth, ensure timely reimbursements to healthcare facilities to send the correct incentives for service delivery. Fifth, invest in fraud minimization strategies such as verification of provider self-assessments reports and claims as well as enrolee membership. This can be implemented by using a risk-based approach to sample facilities for physical verification of self-assessment reports and imposing tough sanctions for providers that are found to present fraudulent self-assessment reports and for those enrolees and providers engaging in fraud. Sixth, harmonize its benefit packages into one benefit package for all its members to reduce inequities in access to needed services. Seventh, simplify the language used in communication of the benefit packages and adopt communication strategies that reach low-income, less educated, rural population groups such as visits to homes and public places such as markets and places of religious worship. Lastly, the NHIF should strengthen monitoring and supervision of healthcare providers and impose sanctions and rewards for quality of care provided.

## Conclusion

Our study shows that while the new reforms sought to expand population and service coverage and reduce OOP, the NHIF remains a passive purchaser due to weaknesses in the design and implementation of the reforms. These weaknesses affected its purchasing actions with negative implications for the health system goals of equity, efficiency and quality. For the reforms to accelerate the country’s progress towards UHC, policy makers from the government (both national and county) as well as the NHIF should put deliberate efforts to align the design and implementation of such reforms with strategic purchasing actions that are aimed at improving equity, efficiency, and quality of health system delivery.

### Supplementary information

**Additional file 1: Appendix 1.** Semi- structured topic Guide for in-depth interviews.

## Data Availability

The datasets generated and/or analysed during the current study are not publicly available as they contain information that could potentially compromise the privacy and confidentiality of the study participants but are available from the corresponding author (RM) on reasonable request.

## Appendix

**Table 9:** Comparison of the benefit packages across schemes

Services	Enhanced schemes: (Civil servants and disciplined services scheme; County schemes, National Police Service and Kenya Prisons Service Medical Scheme [15, 18–20])		National scheme and HISP [23]
Outpatient	Consultation		Consultation
	Laboratory investigations		Laboratory investigations
	Drug administration		Drug administration
	Radiological examinations (basic x-rays)		Radiological examinations (basic x-rays)
	Nursing and midwifery services		Nursing and midwifery services
	Minor surgical procedures		Minor surgical procedures
	Rehabilitation services		rehabilitation services
	Referral for specialised services		Referral for specialised services
	Dental care:	Dental consultation	Dental consultation
		Extractions	Extraction only
		Surgical extractions (including doctors’ fees and theatre costs)	
		Root canal	
		Orthodontics	
		Dental Filling	
		X-rays	
		Dentures	
	Optical services		
	Vaccinations	KEPI vaccines	KEPI vaccines
		Rota virus vaccine	
		Anti-rabies	
		Anti-Snake venom	
		yellow fever vaccine	
	Emergency rescue services:	Local road ambulance	Local road ambulance
		Emergency air rescue	
	Annual medical check-up i. Body mass index ii. Full Haemogram iii. Cholesterol iv. Blood sugar v. Gamma GT vi. Urinalysis vii. PSA (Prostate Specific Antigen for Men above 40) viii. Pap smear for all women ix. Mammogram		
	Optical care		
Specialized services	Renal dialysis		Renal dialysis
	Kidney care package (Renal dialysis and renal transplant)		Kidney care package (Renal dialysis and renal transplant)
	Rehabilitation package: drug and substance abuse		Rehabilitation package: drug and substance abuse
	Oncology: chemotherapy and radiotherapy		Oncology: chemotherapy and radiotherapy
	Radiology: MRI, CT scan		Radiology: MRI, CT scan
	Chronic illness: diabetes and hypertension		Chronic illness: diabetes and hypertension
	Surgical services (Major and minor)		Surgical services (Major and minor)
	Specialised laboratory tests		Specialised laboratory tests
	Overseas treatment for conditions that warrant treatment not available in Kenya		Overseas treatment for conditions that warrant treatment not available in Kenya
	Intensive care and High Dependency Unit		
	Last expense cover		
	In vitro fertilization		
	Hearing aids		
	Prosthetics		

## Abbreviations

CT: Computerized tomography
FGDs: Focus group discussions
FY: Financial Year
IDIs: In-depth interviews
KES: Kenya shillings
MOH: Ministry of Health
MRI: Magnetic Resonance Imaging
NHIF: National Hospital Insurance Fund
OOP: Out-of-pocket
RESYST: Resilient and Responsive Health Systems
UHC: Universal Health Coverage
USD: United States Dollars
