# Supplementary material for: Examining purchasing reforms towards universal health coverage by the National Hospital Insurance Fund in Kenya
Source: Int J Equity Health. 2020 Feb 3;19:19. doi: 10.1186/s12939-019-1116-x (PMC6998279; doi:10.1186/s12939-019-1116-x)
Supplement: Supplementary file 1 — Additional file 1: Appendix 1. Semi- structured topic Guide for in-depth interviews. [file 12939_2019_1116_MOESM1_ESM.docx]

# Additional file 1

**Appendix 1: Semi- structured topic Guide for in-depth interviews**

**Topic Guide for in-depth interviews**

| **Date of discussion:** | **Interviewer:** | |
| --- | --- | --- |
| **Venue:** | **Note taker:** | |
| **Time start:** | **Organization:** | |
| **Time stop:** | **Interviewee’s**  **code:** | |
| **Interview completed** | **Yes** |  |
|  | **No** |  |
| **Reason for Incomplete interview** |  | |
|  |  | |
|  |  | |
|  |  | |

**Interviewer’s remarks about session and issues from debrief**

**Introductory question**

**[Interviewer and note taker to introduce themselves]**

1. Please introduce yourself, your organization, and your organizations mandate
2. Please tell us a little bit about your role in this organization and how long you have been at this role

**REFORM 1: UPWARD REVISION OF NHIF PREMIUM RATES**

- **How was the public** informed of the new NHIF premium rates?
- In your opinion, did the **channels of communication reach each group of the population equally**, or were there groups that had better access to the communication?
  - *PROBE - rich versus poor, rural versus urban, male versus female, adult vs children, young vs elderly, educated vs uneducated, employed vs unemployed*
- What **information was shared** with the public with regards to:
  - The new premium rates?
    - *PROBE: What are the new rates? How do they vary across income groups?*
- How has the **upward revision of premiums affecte**d **citizen’s access** to services:
- *PROBE: What are your thoughts about the affordability of the new NHIF premium rates?*
- *Have enrollment rates changed since the introduction of the new premiums?*
- *Has the rate of default changed?*
- How has the increase in premiums affected the resources available to NHIF?
- *Do the revised premium rates mobilize adequate resources to meet the cost of the benefit packages that it provides to its beneficiaries?*

**REFORM 2: INTRODUCTION OF NEW BENEFIT PACKAGES (outpatient benefit package for the national scheme and special package for all schemes)?**

- What services are offered under the **OUTPATIENT BENEFIT PACKAGE**?
- *How does the outpatient benefit package under the national scheme compare with the outpatient benefit package under the CSS and HISP?*
- What services are offered under the **SPECIAL PACKAGE**?
- *How does the special benefit package compare across the three NHIF schemes (National, CSS and HISP schemes?*
- How were these **benefit packages developed, and what considerations were taken**?
- ***PROBE:*** *Was there an* ***assessment of the health needs, preferences and values*** *of the public and how?*
- *Were the* ***needs of all groups considered equally*** *or did some groups’ preferences and needs receive greater weight?*

***GROUPS*** *- rich vs poor, rural vs urban, male vs female, adult vs children, young vs elderly, educated vs uneducated, employed vs unemployed*

- *What* ***decisions informed*** *the* ***choice of services*** *to be included in t****he outpatient benefit package****? Was this informed by evidence on cost-effectiveness or by burden of disease?*
- *What* ***decisions informed*** *the* ***choice of services*** *to be included in the* ***special package****? Was this informed by evidence on cost-effectiveness or by burden of disease?*
- **How was the public informed** of the introduction of the outpatient benefit package for the National scheme? What about the special packages for all schemes?
- **What information was shared** with the public with regards to these benefit packages?
- ***PROBE****- Do the public know: the contents of the outpatient and the special benefit package, how one becomes eligible or how and where to access the services?*
- In your opinion, **did the channels of communication reach each group of the population equally**, or were there groups that had better access to the communication?
- ***GROUPS*** *- rich versus poor, rural versus urban, male versus female, adult vs children, young vs elderly, educated vs uneducated, employed vs unemployed*
- How are **healthcare providers empaneled** to provide the **outpatient benefit/ special package**?
- *How is this different from existing processes?*
- *Is the process influenced by the method of provider payment e.g. capitation, case-based? (e.g. willingness to accept the pay, capacity to process claims)?*
- How are **healthcare providers contracted** to provide the **outpatient benefit/ special package**?
- *Is there an accreditation process? How has the suspension of the accreditation process affected the quality of services provided to the beneficiaries?*
- *Is there a quality criteria in the contract for the outpatient benefit package? If not, why? In your opinion, how does this affect the quality of services provided?*
- What is the **geographical distribution** of the healthcare providers contracted to provide the **outpatient benefit/ special package** around the country?
- *PROBE: is there a preference for health facilities in certain geographical areas? E.g. rural versus urban? If so, why?*
- *Do the contracted health facilities reach the most vulnerable sections of the community that bear the heavier burden of ill-health?* ***(match with the socioeconomic status of the people when doing document review)***
- What is the **ownership distribution of the contracted** healthcare providers?
- PUBLIC vs PRIVATE? *Is there a preference for one over the other? If so, why?*
- *Does* ***ownership affect*** *the range of services available to a beneficiary for the outpatient benefit package and the special package?*
- What is the **facility level distribution** of the contracted healthcare providers?
- *PRIMARY vs SECONDARY vs TERTIARY? Is there a preference for certain levels over others? If so, why?*
- *Does* ***level of facility affect*** *the range of services available to a beneficiary for the outpatient benefit package and the special package?*
- Do the contracted health facilities have **the structural capacity** (e.g. sufficient health workers, medicines, lab facilities, outpatient facilities) to provide the **outpatient benefit package/ special package?**
- *Does service delivery infrastructure for these benefit packages* ***vary between similar types or levels*** *of contracted health facilities? If so, how and why?*

*TYPES- private/public; LEVELS- primary/secondary/tertiary health facilities*

- *Does service delivery infrastructure for these benefit packages* ***vary across geographical areas*** *in the country? (Rural versus urban)*
- *Has the government invested in the structural capacity of healthcare providers to facilitate the provision of the outpatient benefit and the special package?*
- How has the introduction of the new benefit packages **affected citizen’s access** to services?
- ***PROBE-*** *Does access to the* ***outpatient benefit /special package*** ***vary across the beneficiaries*** *in the three NHIF schemes? If so, how and why?*
- How **does the NHIF obtain information from healthcare** providers on the provision of these benefit packages?
- *Are these reports available (ask them to provide you with copies if regulations allow sharing)?*
- *PROBE: What is your opinion on the adequacy of provider reporting on the utilization of services provided under these packages?*
- Does the NHIF **monitor the quality of services** under these benefit packages? If yes, how?
- What are the **performance-based rewards or sanctions** associated with the quality of care provided for these services?
- In your opinion, does the NHIF **have adequate resources** to provide t**hese benefit packages**? If yes/ no, how and why?
- Do you think the **introduction of these benefit packages** has created opportunities for fraud by NHIF?

**REFORM 3: INTRODUCTION OF NEW PROVIDER PAYMENT METHODS AND RATES (**

**a) Capitation payment method for the outpatient benefit package, b) case-based payment for the special package, and c) increase in inpatient per diem rate)**

- How was the **capitation rate for the outpatient benefit package** determined?
- *PROBE: Were they based on any actuarial and/ or costing analysis?*
- *Were the healthcare providers consulted?*
- *Do these payments approximate the actual average costs of providing services in the outpatient benefit package?*
- How were the **case-based payments for the special package** determined?
- *PROBE: Were they based on any actuarial and/ or costing analysis?*
- *Were the healthcare providers consulted?*
- *Do these payments approximate the actual average costs of providing services in the special package?*
- How were the **new inpatient per diem rates** determined?
- *PROBE: Were they based on any actuarial and/ or costing analysis?*
- *Were the healthcare providers consulted?*
- *Do these payments approximate the actual average costs of providing inpatient services?*
- How do these **provider payment methods and rates** compare **across the three different NHIF schemes**?
- *PROBE- Are the rates similar or different? How and why?*
- In your opinion, **how do differences (if any)** **affect access** to the services under the **outpatient, special or inpatient package** by beneficiaries across the three schemes?
- How have the **new provider payment methods** (capitation, case-based payment and new per diem rates) **affected the empanelment process** of healthcare providers?
- *How have they affected the licensing and accreditation of healthcare providers?*
- *Is there a preference for certain* ***types*** *(public/private) or* ***levels*** *(Primary / secondary/ tertiary) of healthcare facilities? If so, why?*

- **What is the influence** of these new provider payment methods on **co-payments and balance billing for the beneficiarie**s? How has this affected access to the benefits?
- What is the **influence of these new provider payment methods** on **cost-management or cost-containment** by **healthcare providers**?
- *Is there a preference for lower-cost over higher-cost treatment, preventive vs curative or upward referral of patients?*
- What is the **influence of these new provider payment methods** on the **quality of services provided**?
- Do the **new provider payment methods take into consideration the quality of services provided**? If so, how?
- How and when are the capitation and case-based payments for the new benefit packages made to the healthcare providers?
- *Probe: are these payments made in a timely manner?*
